# MicroRNA-214 Promotes Apoptosis in Canine Hemangiosarcoma by Targeting the COP1-p53 Axis

**DOI:** 10.1371/journal.pone.0137361

**Published:** 2015-09-03

**Authors:** Kazuki Heishima, Takashi Mori, Hiroki Sakai, Nobuhiko Sugito, Mami Murakami, Nami Yamada, Yukihiro Akao, Kohji Maruo

**Affiliations:** 1 United Graduate School of Veterinary Sciences, Gifu University, Gifu, Gifu, Japan; 2 Department of Veterinary Clinical Oncology, Faculty of Applied Biological Sciences, Gifu University, Gifu, Gifu, Japan; 3 Department of Veterinary Pathology, Faculty of Applied Biological Sciences, Gifu University, Gifu, Gifu, Japan; 4 United Graduate School of Drug Discovery and Medical Information Sciences, Gifu University, Gifu, Gifu, Japan; Colorado State University, UNITED STATES

## Abstract

MicroRNA-214 regulates both angiogenic function in endothelial cells and apoptosis in various cancers. However, the regulation and function of miR-214 is unclear in canine hemangiosarcoma, which is a spontaneous model of human angiosarcoma. The expression and functional roles of miR-214 in canine hemangiosarcoma were presently explored by performing miRNA TaqMan qRT-PCR and transfecting cells with synthetic microRNA. Here, we report that miR-214 was significantly down-regulated in the cell lines used and in clinical samples of canine hemangiosarcoma. Restoration of miR-214 expression reduced cell growth and induced apoptosis in canine hemangiosarcoma cell lines through transcriptional activation of p53-regulated genes although miR-214 had a slight effect of growth inhibition on normal endothelial cells. We identified COP1, which is a critical negative regulator of p53, as a novel direct target of miR-214. COP1 was overexpressed and the specific COP1 knockdown induced apoptosis through transcriptional activation of p53-regulated genes as well as did miR-214-transfection in HSA cell lines. Furthermore, p53 knockdown abolished the miR-214-COP1-mediated apoptosis; thus, miR-214 and COP1 regulated apoptosis through controlling p53 in HSA. In conclusion, miR-214 functioned as a tumor suppressor in canine hemangiosarcoma by inducing apoptosis through recovering the function of p53. miR-214 down-regulation and COP1 overexpression is likely to contribute to tumorigenesis of HSA. Therefore, targeting miR-214-COP1-p53 axis would possibly be a novel effective strategy for treatment of canine hemangiosarcoma and capable of being applied to the development of novel therapeutics for human angiosarcoma.

## Introduction

Neoplastic endothelial proliferative diseases such as human angiosarcoma (AS) and canine hemangiosarcoma (HSA) are serious diseases for both humans and dogs. AS usually occurs in the skin, especially the scalp and face of elderly people [1], as well as in the female breast [2], which sarcomas arising at this site are usually resulted from radiation therapy [3]. Other primary sites are also reported to be the liver [4] and spleen [5]; however, the incidence in those sites are less frequent than that of dermal AS [6]. The prognosis of AS is poor because of the frequent local recurrence and distant metastasis. The overall 5-year survival rate is approximately 43 to 60% with the median survival being 7 months [6,7]. However, AS has not been well studied compared to the other major tumors because of its low incidence; i.e., it accounts for only 1.8% of soft-tissue sarcomas [8]. The lack of case number and research modality for AS makes it difficult to understand the detailed pathobiology of AS and to develop a new strategy for the treatment despite the poor prognosis. HSA, a canine malignant endothelial neoplasm, shares many features with AS in terms of malignant behaviors such as the low survival rate and frequent metastasis. Surgery and doxorubicin-based chemotherapy increase survival duration; however, the 1-year survival rate is less than 10% [9]. Unlike in the case of AS, HSA is relatively common, accounting for approximately 20% of all canine soft-tissue sarcomas [10]. Although the most common primary site of HSA is the spleen, HSA occurs in various organs such as liver, right atrium of heart and skin with much higher incidence than that in humans [11]. Domestic dogs share environmental factors with humans and develop HSA spontaneously with high incidence. Furthermore, the clinical and pathologic similarities between AS and HSA make dogs a valuable resource for the development of therapeutics for AS [12]. Although the direct translation of results from HSA to AS study should be careful because only a few comparisons between these sarcomas have been made at the cell signaling level, the study of HSA have potential to provide a powerful model for AS.

MicroRNAs (miRNAs) are short noncoding RNAs conserved among species, and they are master-regulators of diverse biological processes including tumorigenesis and angiogenesis. Anti-oncomirs, a class of miRNAs that suppress oncogenes [13], are commonly down-regulated in malignant tumors. Furthermore, some miRNAs also play indispensable roles in angiogenesis and vascular development [14]. miR-214 is one of the miRNAs regulating both tumorigenesis and angiogenesis [15,16]. miR-214 is down-regulated and works as an anti-oncomir in breast cancer [17], cervical cancer [18,19], hepatocellular carcinoma [20], hepatoma [21], cholangiocarcinoma [22], colorectal carcinomas [23,24], glioma [25], multiple myeloma [26], prostate cancer [27], bladder cancer [28,29], and rhabdomyosarcoma [30]. Moreover, miR-214 is abundantly expressed in EC, negatively controlling angiogenic function in main vascular cell types and tissue with abundant vasculature [16,31,32]. However, the regulation and function of miR-214 in the malignant endothelial proliferative diseases are not well understood. Clarification of the regulation and pathobiological function of miR-214 should contribute to a better understanding of the detailed molecular pathogenesis and to the development of new therapeutics for those endothelial proliferative diseases.

p53, a well-established tumor suppressor, is a stress-activated transcriptional factor. p53 activates the transcriptions of *CDKN1A*, *FAS*, *BAX* and *THBS1* genes, leading to growth arrest, apoptosis or anti-angiogenesis action, thereby preventing cells from replicating a genetically abnormal genome [33]. Although p53 is frequently mutated in human and canine malignancies [34,35], recent studies showed that p53 mutations are rare in HSA [36] as well as in AS [37]. However, such findings do not indicate that the p53 pathway is intact in those tumors. p53 is rapidly turned over by proteasomal degradation pathways such as those utilizing E3 ubiquitin ligases. MDM2, which is one of the E3 ubiquitin ligases targeting p53 [38], is reportedly overexpressed in AS [39], suggesting that nullification of p53 by these E3 ubiquitin ligases might be important in AS and HSA rather than the p53 gene mutation itself. COP1 E3 ubiquitin protein ligase (also known as RFWD2) is one of the other critical negative regulators of p53 [40]. COP1 is over-expressed and acts as a negative regulator of p53 via ubiquitination in several cancers such as hepatocellular carcinoma [41], and breast and ovarian adenocarcinomas [42]; and thus COP1 is considered to be a promising target for treatment of those cancers [41].

In this study, we examined the expression and function of miR-214 in HSA and found that miR-214 was significantly down-regulated in both HSA clinical samples and cell lines. Our data indicated that miR-214 exerted apoptosis through transcriptional activation of p53. Furthermore, we identified COP1 E3 ubiquitin ligase as a novel direct target of miR-214, which ligase is the molecule responsible for miR-214-mediated apoptosis. COP1 is overexpressed in HSA cell lines and the knockdown of this ligase induced p53-dependent apoptosis in HSA cell lines.

## Material and Methods

### Clinical samples

Primary splenic HSA tissues (n = 7) were obtained from dogs diagnosed as having HSA at the Animal Medical Center of Gifu University. All animals were histopathologically diagnosed as HSA by at least one JCVP board-certified pathologist. Normal splenic tissues (2 tissues from dogs with HSA and 5 tissues from other dogs) were obtained as control tissues. All samples were stabilized by use of RNA*later* Solutions (Ambion, Life Technologies, Thermo Fisher Scientific, Waltham, MA, USA) and stored at-80°C. Information about the dogs is given in S1 Table. All clinical samples were collected from a portion of biopsy for diagnosis. The sampling and application for this study of all clinical materials were carried out with the owner's consent based on the guidelines that were approved by the Committee of Animal Medical Center of Gifu University. The owners provided their written informed consent for clinical samples from their dogs to be collected for this study.

### Cell lines and culture conditions

The HSA cell lines used, JuB2, Re12, and Ud6 originated from hepatic, atrial, and splenic HSA, respectively [43], and were cultured in D-MEM supplemented with 10% FBS (Sigma-Aldrich, St. Louis, MO, USA). Canine aortic endothelial cells (CnAOEC), used as normal canine primary EC, were purchased from Cell Applications Inc. (San Diego, CA, USA) and cultured in canine endothelial cell basal medium with growth supplement.

### Transfection with small RNAs

All HSA cell lines and CnAOEC for transfection with small RNAs were seeded in 6-well plates at the concentration of 0.5x10^5^ per well on the day before the transfection. miRNA-214 (1 or 10 nmol/L; mirVana miRNA mimic; Ambion), miRNA-214 inhibitor (1 nmol/L; mirVana miRNA inhibitor; Ambion) or 10 nmol/L of non-specific control miRNA (sequence: 5'-GUA GGA GUA GUG AAA GGC C-3', Hokkaido System Science Co., Ltd., Hokkaido, Japan) was used for transfection of cells with Lipofectamine RNAi-MAX (Invitrogen, Carlsbad, CA, USA) according to the manufacturer's instructions. Short interfering RNA (siRNA) for canine *COP1* (siR-cop1, sense: CCA AGU UUA CUC GGU AUA Ad TdT, antisense: UUA UAC CGA GUA AAC UUG GdT dT) and *TP53* (siR-tp53, sense: GGA CGA CAG AAA CAC UUU Utt, antisense: AAA AGU GUU UCU GUC GUC Cag) were newly designed and purchased from Sigma-Aldrich or Life Technologies. These siRNA are transfected in the same way as miRNA transfection. The transfection rate in this study was confirmed to be over 90% by microscopic examination using GFP-tagged plasmid. After incubation for 72 hours, the transfected cells were harvested for intended analyses.

### Cell proliferation and viability assays

Cell proliferation and viability was examined by using the 3-(4,5-dimethylthiazol-2-yl)-2,5- diphenyltetrazolium bromide (MTT) assay. HSA cells or CnAOEC were seeded in 12-well plates. The cells were transfected with miR-214 or control RNA as described above. After 72 hours, MTT-1 labeling reagent (Roche Applied Science, Basel, Switzerland) was added to the medium (1:10); and the cells were then incubated at 37°C for 4 h. Next, the same amount of solublization solution was added; and incubation was continued overnight in a humidified atmosphere (37°C, 5% CO_2_). Subsequently, the plates were shaken thoroughly, and 570 nm absorbance of the wells was measured with iMark Microplate Absorbance Reader (Bio-Rad, Hercules, CA, USA), having a reference filter of 630 nm. The values are calibrated by the values of blank wells.

### Hoechst 33342 nuclear staining

Nuclear fragmentation was assessed by Hoechst 33342 staining. In brief, after transfection with small RNAs, the cells were incubated with 5 mg/mL of Hoechst 33342: bisBenzimide H 33342 trihydrochloride (Sigma-Aldrich) at 37°C for 30 min. After the cells had been washed with cold PBS twice, nuclear morphology was visualized by using a fluorescence microscope (Olympus, Shinjuku, Tokyo, Japan). The percentage of apoptotic cells was calculated by counting the number of cells with fragmented nuclei among a total of 500 cells.

### Annexin V and PI double staining

Translocation of PS in the plasma membrane was measured by using Annexin V Alexa Fluor 488 and propidium iodide double staining (Tali Apoptosis Kit, Life Technologies) according to the manufacturer’s procedure.

### Cell cycle analysis

Cell cycle progression was analyzed by quantification of cellular DNA content using PI staining (Tali Cell Cycle Kit, Life Technologies) according to the manufacturer’s procedure.

### RNA extraction and quantitative real-time polymerase chain reaction analysis

Total RNA was extracted from the cells by using NucleoSpin miRNA (MACHEREY-NAGEL, Düren, Deutschland) according to the standard protocol. RNA concentration and purity were assessed by UV spectrophotometry. RNA integrity was checked by formaldehyde gel electrophoresis. For detection of the mature miR-214 expression levels (sequence: 5'-ACA GCA GGC ACA GAC AGG CAG U-3'), a miRNA quantitative reverse transcription polymerase chain reaction (miRNA qRT-PCR) assay was performed. Total RNA (25 ng) was reverse transcribed to cDNA by using a TaqMan MicroRNA Reverse Transcription Kit (Applied Biosystems, Thermo Fisher Scientific). Subsequently, mature miR-214 expression levels were assessed by performing TaqMan MicroRNA Assays (AB Assay ID 002306, Applied Biosystems). The relative expression of mature miR-214 level was normalized to the RNU6B endogenous control (AB Assay ID 001093, Applied Biosystems) [44,45] and determined by the 2^-ΔCt^ method. Each measurement was performed in triplicate. For determination of mRNA expression, quantitative RT-PCR (qRT-PCR) was performed. Total RNA (0.5 μg) was reverse transcribed to cDNA by use of a PrimeScript RT Reagent Kit (Takara Bio Inc., Shiga, Japan). SYBR green qRT-PCR was performed with Universal SYBR Select Master Mix (Applied Biosystems) and TaKaRa Thermal Cycler Dice Real Time System II (Takara) with gene-specific primers (see S2 Table). Relative expression levels were normalized to TATA box binding protein (*TBP*), which is stably expressed in canine cells and used as internal control for qRT-PCR [46]. Relative gene expression was calculated by using the 2^-ΔCt^ method.

### Immunoblotting

Whole cell lysates were prepared by using RIPA buffer containing Protease Inhibitor Cocktail (Sigma-Aldrich) and Phosphatase Inhibitor Cocktail (nakarai tesque, Kyoto, Japan). Protein concentrations were measured by the Bradford protein assay (DC Protein assay kit, Bio-Rad). Protein samples (5 μg/lane) were subjected to 10–15% sodium dodecylsulphate-polyacrylamide gel electrophoresis (SDS-PAGE) and transferred to 0.45 μm polyvinylidene fluoride membranes (Immobilon-P Membrane, EMD Millipore). After blockage of nonspecific reactions for 1 hour with 5% nonfat milk in PBS containing 0.1% Tween 20 (TBS-T), the membrane was incubated overnight at 4°C with a given primary antibody. The primary antibodies used for immunoblotting were anti-Cleaved Caspase-3 (Asp175) rabbit monoclonal antibody (clone 5A1E, Cat. #9664, Cell Signaling Technology, Danvers, MA, USA), anti-PARP rabbit polyclonal antibody (Ab-2; Cat. #RB-1516, Thermo Fisher Scientific), anti-COP1 mouse monoclonal antibody (clone 1E4, Cat. #WH0064326M1, Sigma-Aldrich), and anti-p53 mouse monoclonal antibody (clone 80/p53, Cat. #610183, BD Biosciences, Franklin Lakes, NJ, USA). The membranes were then washed and subsequently incubated at room temperature with the secondary horseradish peroxidase-linked anti-mouse IgG or anti-rabbit IgG antibodies (Cell Signaling Technology) diluted 1:1000 and then washed 3 times with TBS-T. The immunoblots were visualized by using Luminata Forte Western HRP substrate (EMD Millipore). The loaded amount was verified by re-incubating the same membrane with anti-β-actin mouse monoclonal antibody (clone AC-74, Cat. #A5316, Sigma-Aldrich).

### Computational microRNA target and target site prediction

Two different databases (TargetScan Release 6.2 http://www.targetscan.org/ [47] and miRDB http://mirdb.org/ [48,49]) were used to identify potential miR-214 target genes and target sites.

### Luciferase reporter assay

Luciferase reporter vectors of wild-type, mutant-1 and mutant-2 reporter vectors were synthesized. The sequence region including the putative binding sites of miR-214 (21–23 and 31–38 in the 3'UTR of COP1/RFWD2 mRNA, XM_537181.4) was amplified by RT-PCR with TaKaRa Ex Taq (Takara) and TaKaRa PCR Thermal Cycler Dice (Takara). Subsequently, the wild-type sequence was inserted into a pMIR-REPORT Luciferase miRNA Expression Reporter Vector (Applied Biosystems) according to the manufacturer’s protocol. The mutant reporter vectors, including mutant-1 and mutant-2, were synthesized by using a PrimeSTAR Mutagenesis Basal Kit (Takara). The mutation sites and sequences of the vector were confirmed by sequence analysis.

For luciferase assays, Ud6 cells were seeded in 6-well plates at a concentration of 0.5×10^5^ per well on the day before the transfection. The cells were transfected with 0.1 μg of the above vectors and co-transfected with 10 nM miR-214 or control RNA by use of Lipofectamine RNAiMAX. After 24 hours, the transfected cells were collected, and their firefly and renilla luciferase activities were measured with a Dual-Glo Luciferase Assay System (Promega, Fitchburg, WI, USA) according to the manufacturer’s protocol. Measurements were performed with a GloMax 20/20 Luminometer (Promega). Firefly luciferase activity was normalized to Renilla luciferase activity.

### Statistical analysis

Differences were statistically evaluated by the Mann-Whitney test or the unpaired two-tailed t-test in each examination. A p-value of less than 0.05 was considered to be significant.

## Results

### miR-214 was down-regulated in HSA cell lines and clinical samples

**Fig 1:**
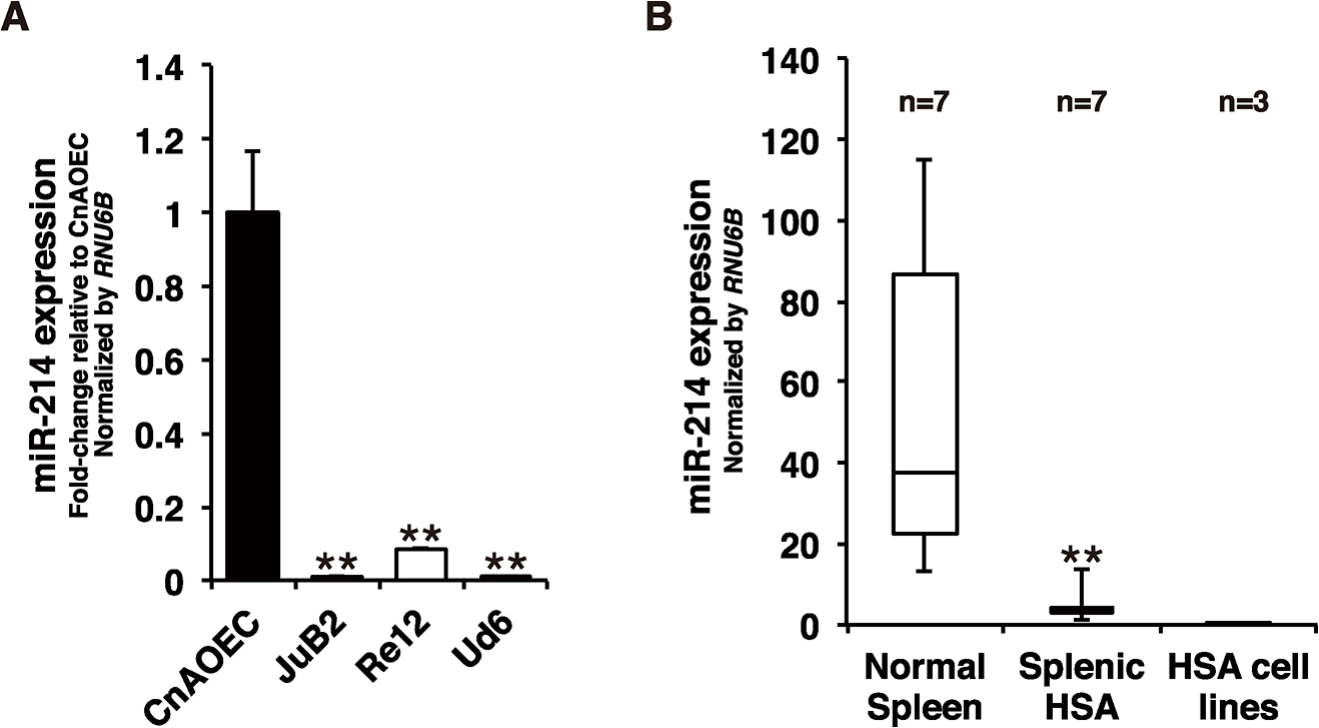
miR-214 is down-regulated in HSA cell lines and clinical samples. (A) Quantitative measurement of miR-214 revealed that miR-214 expression was significantly down-regulated in HSA cell lines (JuB2, Re12 and Ud6) compared with that in normal EC (CnAOEC). All data are presented as the mean of triplicate experiments with error bars indicating the s.d. (Two-tailed t-test; **p<0.01 for comparisons to miR-214 expression of CnAOEC). (B) The expression of miR-214 was significantly down-regulated in splenic HSA tissues compared with that in normal spleen tissues. The fold change of the median was 0.10051. (Mann-Whitney test; **p<0.01 for comparison between the expression of miR-214 in splenic HSA tissues and that in normal spleen tissues)

miR-214 plays important roles in regulating the angiogenic function of endothelial cells [16,32]. Moreover, miR-214 is down-regulated and has been implicated in tumorigenesis of certain human cancers [17,22,26,30]. We hypothesized that miR-214 plays important roles in HSA and contributes to the tumorigenesis of HSA. We first assessed the expression levels of miR-214 in HSA cell lines established from diverse primary sites (JuB2 from hepatic HSA, Re12 from atrial HSA, and Ud6 from splenic HSA) and in normal primary EC (CnAOEC) by performing miRNA TaqMan qRT-PCR. We found that miR-214 was significantly down-regulated in all HSA cell lines tested regardless of the primary sites of origin of the cell lines (Fig 1A). Next, we examined the expression of miR-214 in clinical samples of splenic HSA (n = 7) and normal splenic tissues (n = 7), also by miRNA TaqMan qRT-PCR. Consistent with the results obtained with the cell lines, miR-214 was also significantly down-regulated in splenic HSA tissues compared with its expression in the normal splenic tissues (Mann-Whitney test; p = 0.00328; Fig 1B). These results indicate that miR-214 was down-regulated in HSA, suggesting broad and essential roles of miR-214 in the pathogenesis of HSA.

### Ectopic expression of miR-214 induced apoptosis in HSA cell lines

**Fig 2:**
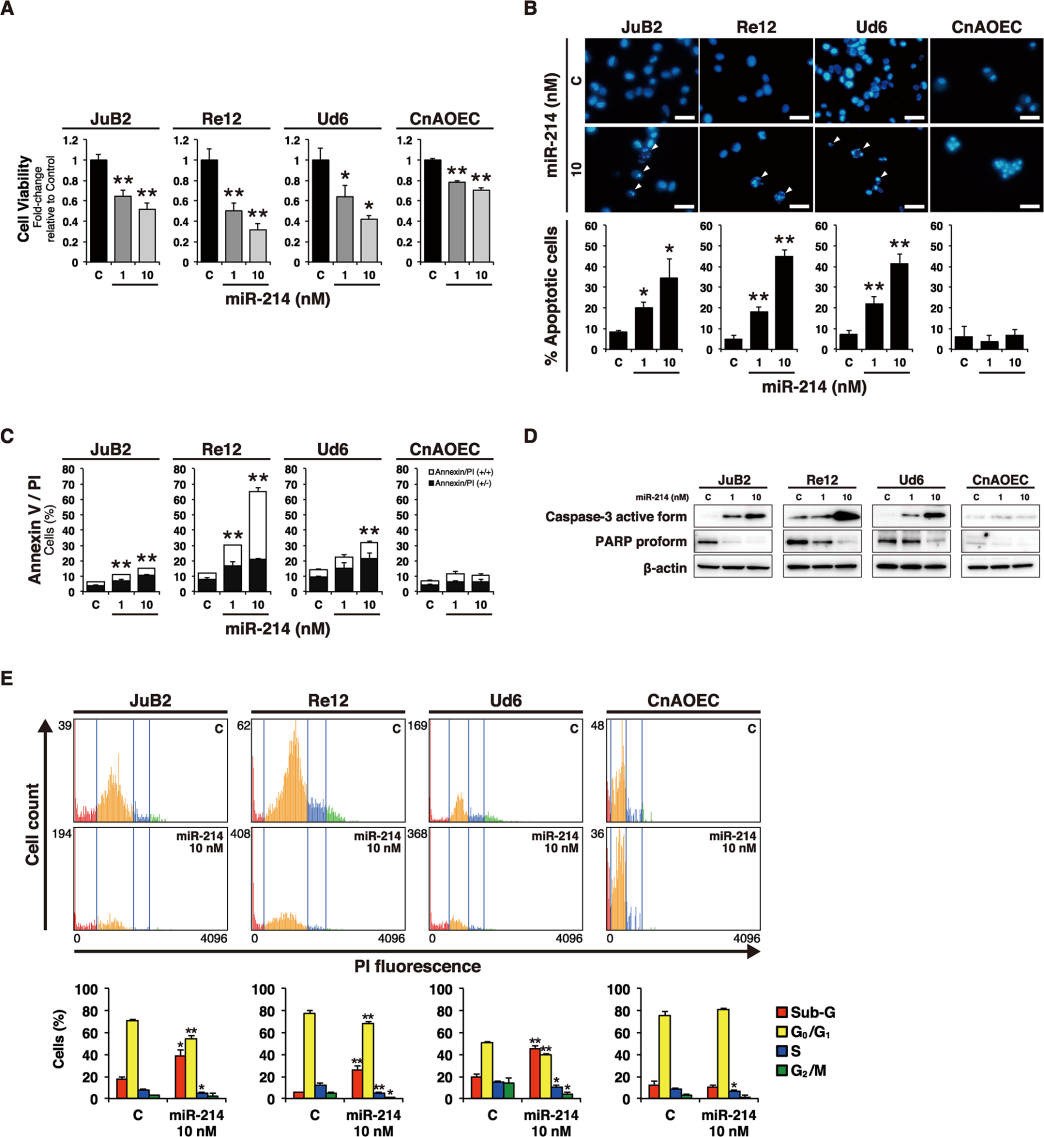
miR-214 decreased the number of viable cells and induced apoptosis in HSA cell lines. (A) Cell viability was assessed by performing the MTT assay. miR-214-transfection decreased the number of viable cells in HSA cell lines and that of control EC. However, the degree of growth inhibition was slight for the control EC compared with that for the HSA cell lines. All data are presented as the mean of triplicate experiments with error bars indicating the s.d. (Two-tailed t-test; *p<0.05, **p<0.01). (B) Morphological assessment of nuclei by Hoechst 33342 nuclear staining. miR-214 increased the number of cells with fragmented nuclei (white arrow heads) dose-dependently in HSA cell lines, whereas it caused no morphological changes in the nuclei of the control EC. Scale bars in the photographs indicate 25 μm. The graph shows the percentage of cells with fragmented nuclei among a total 500 cells. All data are presented as the mean of triplicate experiments with error bars indicating the s.d. (Two-tailed t-test; *p<0.05, **p<0.01). (C) Apoptotic cell count by Annexin V/ PI double staining. Annexin V-positive/ PI-negative and Annexin V-positive/ PI-positive cells represent early and late-phase apoptosis, respectively. miR-214 increased both early and late-phase apoptosis in HSA cell lines. However, miR-214 hardly induced apoptosis in the control EC. All data are presented as the mean of triplicate experiments with error bars indicating the s.d. The statistical significances stated were referred to the entire Annexin/PI population (Two-tailed t-test; *p<0.05, **p<0.01). (D) Immunoblotting for caspase-3 active form and PARP proform. miR-214 increased the amount of the active form of caspase-3 and cleavage of PARP proform dose-dependently in HSA cell lines (JuB2, Re12 and Ud6) although significant changes were not observed in control EC (CnAOEC) transfected by miR-214. β-actin was used for normalization of the amount of sample loaded. (E) Cell cycle analysis. miR-214 increased the sub-G fraction and decreased S and G_2_/M fractions in all HSA cell lines, indicating that miR-214 induced apoptosis in HSA cell lines; however, only slight decrease of S fraction were observed in control EC. The histograms show the representative data of each cell line transfected by 10 nM of miR-214 or control RNA in the triplicated analysis. The data of bar graphs are presented as the mean of triplicate experiments with error bars indicating the s.d. (Two-tailed t-test; *p<0.05, **p<0.01).

To investigate whether miR-214 functionally affected HSA cells, we transfected miR-214 in 3 HSA cell lines (JuB2, Re12, and Ud6) and a normal EC (CnAOEC) with this miRNA. As a result, ectopic expression of miR-214 induced a dose-dependent growth inhibition in these HSA cell lines; whereas miR-214 was only slightly inhibitory toward the growth of the normal control EC at 72 hours post-transfection (Fig 2A and S2 Fig).

In order to examine whether miR-214 induced cell death of HSA cells and EC, we conducted 4 different experiments, i.e., Hoechst 33342 nuclear staining, immunoblotting for Caspase-3 active form and PARP, Annexin V/ PI double staining, and cell cycle analysis, to assess this possibility. Hoechst 33342 nuclear staining showed increased nuclear-fragmentation in miR-214-transfected HSA cells (Fig 2B). Additionally, Annexin V/ PI double staining revealed an increased percentage of Annexin V/ PI (+/-) and (+/+) cells, indicating an increase in the number of early- and late-phase apoptotic cells in miR-214-transfected HSA cells (Fig 2C). Biochemically, immunoblotting showed cleaved caspase-3 and cleavage of the PARP proform, which is the substrate of activated caspase-3, in the miR-214-transfected HSA cells (Fig 2D), indicating the activation of caspase-3 in the apoptotic processes. Cell cycle analysis revealed that increased sub-G fraction, which indicates nuclear fragmentation by apoptosis in miR-214-transfected HSA cells (Fig 2E). However, such apoptotic changes were hardly (Fig 2B and 2E) or slightly (Fig 2C and 2D) observed in the miR-214-transfected control EC, which finding indicates that miR-214 had growth inhibitory effects on rather than induced apoptosis in normal EC. These results indicate that the ectopic expression of miR-214 induced apoptosis in HSA cells and that miR-214 had a slight growth inhibiting effect on normal EC, suggesting that miR-214 could function as an anti-oncomir in HSA cells.

### miR-214 exerted up-regulation of p53-regulated genes in HSA cells

**Fig 3:**
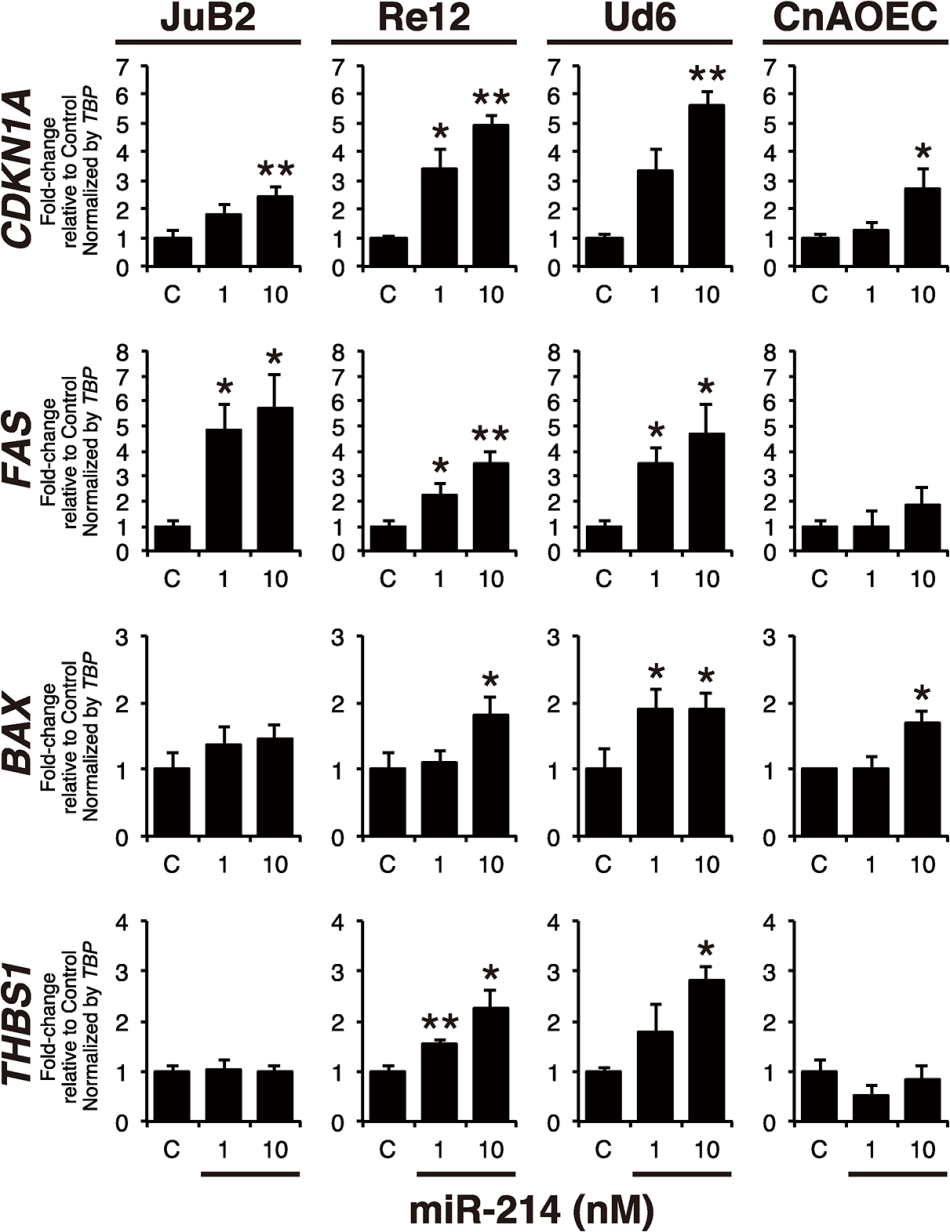
miR-214 provoked expression of p53-regualted genes. The expression of p53-regulated genes including *CDKN1A*, *FAS*, *BAX* and *THBS1* in miR-214 transfected samples was assessed by qRT-PCR. The expression of p53-regulated genes was up-regulated dose-dependently in all HSA cell lines transfected with 1 nM or 10 nM of miR-214; however, only slight effects were observed in normal EC. *TBP* was used for the normalization of each mRNA expressions. All data are presented as the mean of triplicate experiments with error bars indicating the s.d. (Two-tailed t-test; *p<0.05, **p<0.01). Taken together, the above data indicate that miR-214 induced apoptosis through transcriptional activation of p53-regulated genes.

The above results showed that miR-214 exhibited its growth inhibitory effect mainly by inducing apoptosis in HSA cells. For further exploration of miR-214-induced apoptosis, we focused on p53, because the dysregulation of p53 in HSA and AS patients was suggested in previous reports [36,37]. p53 acts as a transcriptional factor for the expression of p53-regulated genes such as *CDKN1A*, *BAX*, *FAS* and *THBS1*. Therefore, we examined the mRNA expression levels of p53-regulated genes by mRNA qRT-PCR to elucidate the effects of miR-214 on the function of p53 and found that the introduction of miR-214 significantly increased the p53-regulated gene expression in all HSA cell lines although the degree of up-regulation differed between each line; while these effects were slight in control EC (Fig 3). These results indicate that miR-214 played a role in cell growth, death and angiogenesis by up-regulating p53-regulated genes in HSA cell lines.

### COP1 is a direct target of miR-214

**Fig 4:**
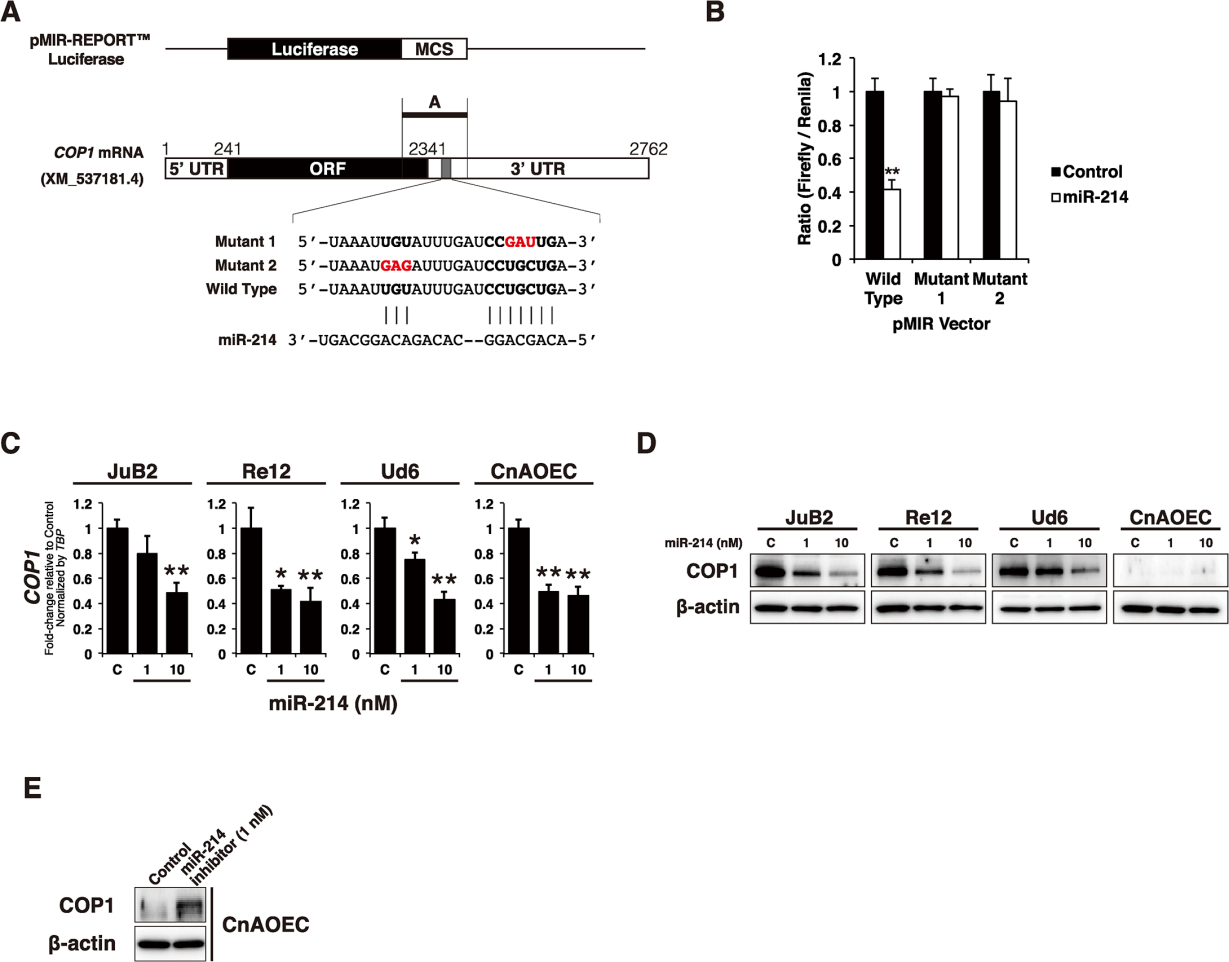
COP1 was a direct target of miR-214. (A) Schematic diagram of luciferase reporter vectors. The sequence region including the putative binding sites of miR-214 (21–23 and 31–38 in the 3'UTR of *COP1* mRNA, XM573181.4) was inserted in the pMIR-REPORT Luciferase vector as wild type. Two types of mutant vectors (mutant-1 and mutant 2) are also synthesized as shown in this figure. (B) Luciferase reporter assay for effect of co-transfection with miR-214 on luciferase activity in Ud6 cells. miR-214 was able to repress the luciferase activity of Ud6 cells transfected with the wild-type vector although it failed to show repression when mutant-1 and mutant-2 vectors were used for the co-transfection, indicating that miR-214 directly targeted *COP1* mRNA through binding to the predicted seed sequences in 3'UTR of *COP1* mRNA. All data are present as the mean of triplicate experiments with error bars indicating the s.d. (Two-tailed t-test; *p<0.05, **p<0.01). (C) Effect of transfection of HSA cell lines and normal EC with miR-214 on *COP1* mRNA expressions in them, assessed by qRT-PCR. *COP1* mRNA expression was down-regulated dose-dependently by miR-214-transfection. *TBP* was used for the normalization of each mRNA expressions. All data are expressed as the mean of triplicate experiments with error bars indicating the s.d. (Two-tailed t-test; *p<0.05, **p<0.01). (D) Expression level of COP1 protein in HSA cell lines and normal EC transfected with miR-214. COP1 protein expression was also dose-dependently down-regulated in the HSA cell lines transfected with miR-214. (E) Effect of miR-214-knockdown in control EC. Knockdown of miR-214 up-regulated COP1 expression in control EC.

The fact that miR-214 evokes transcriptional activation of p53-regulated genes suggests that the direct target of miR-214 possibly regulated p53 transcriptional activity. To get insight into the mechanisms by which miR-214 controlled the expression of p53-regulated genes, we searched for putative p53-related mRNA targets of miR-214 by using TargetScan [47] and miRDB [48,49]. Thereby, we identified COP1 E3 ubiquitin protein ligase (also known as RFWD2), a negative regulator of p53, as a possible target of miR-214, which target site is conserved in both human and canine (S1A and S1B Fig). To investigate whether miR-214 could bind to the predicted seed sequences in the 3' UTR of *COP1* mRNA, we conducted a luciferase reporter assay. The wild-type *COP1* 3'UTR luciferase reporter, containing a conserved target site (Fig 4A), showed significantly reduced luciferase activity after co-transfection with miR-214 (Fig 4B). Moreover, mutant 1 and 2 vectors, which contained a mutated target sequence complementary to the miR-214 seed region (Fig 4A), relieved the repressive effect of miR-214 on the luciferase activity (Fig 4B).

To investigate whether miR-214 was able to regulate *COP1* expression, we conducted mRNA qRT-PCR and immunoblotting for determining the expression of COP1 mRNA and protein in miR-214-transfected HSA cell lines. Our data showed that the ectopic expression of miR-214 down-regulated COP1 expression at both mRNA (Fig 4C) and protein (Fig 4D) levels. Furthermore, miR-214-knockdown by the inhibitor up-regulated COP1 expression in normal EC (Fig 4E), suggesting that miR-214 is a key regulator of COP1. Taken together, these results demonstrated that miR-214 directly targeted *COP1* mRNA through binding to the predicted seed sequence in the 3'UTR of *COP1* mRNA and thereby negatively regulated COP1 expression at both mRNA and protein levels.

### COP1 was overexpressed in HSA cell lines

**Fig 5:**
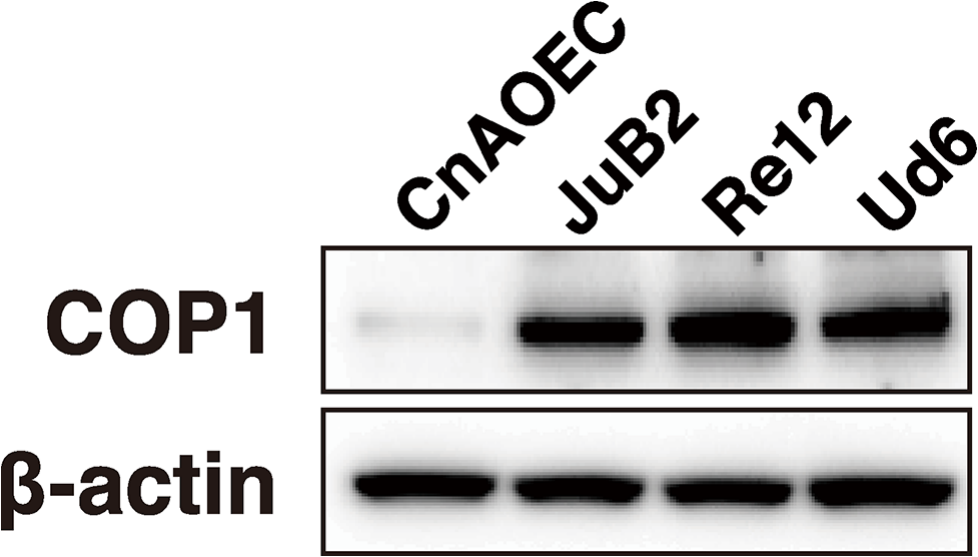
COP1 was overexpressed in HSA cell lines. The protein expression of COP1 in HSA cell lines and normal EC assessed by immunoblotting. COP1 was strongly expressed in HSA cell lines, however, normal EC weakly expressed COP1.

Because miR-214 directly regulated COP1 expression, we hypothesized that down-regulation of miR-214 induced up-regulation of COP1 in HSA. In order to validate the differences of COP1 expression, we assessed the expression of COP1 protein in the HSA cell lines and control EC by immunoblotting. Consistent with our hypothesis, COP1 protein was overexpressed in HSA cell lines, whereas normal EC weakly expressed COP1 (Fig 5). The result implies that miR-214 down-regulation possibly leads to COP1 overexpression in HSA; therefore, miR-214-dependent COP1 overexpression is a key event for dysregulated p53 expression in HSA patients.

### COP1 knockdown induced apoptosis and up-regulation of p53-regulated genes

**Fig 6:**
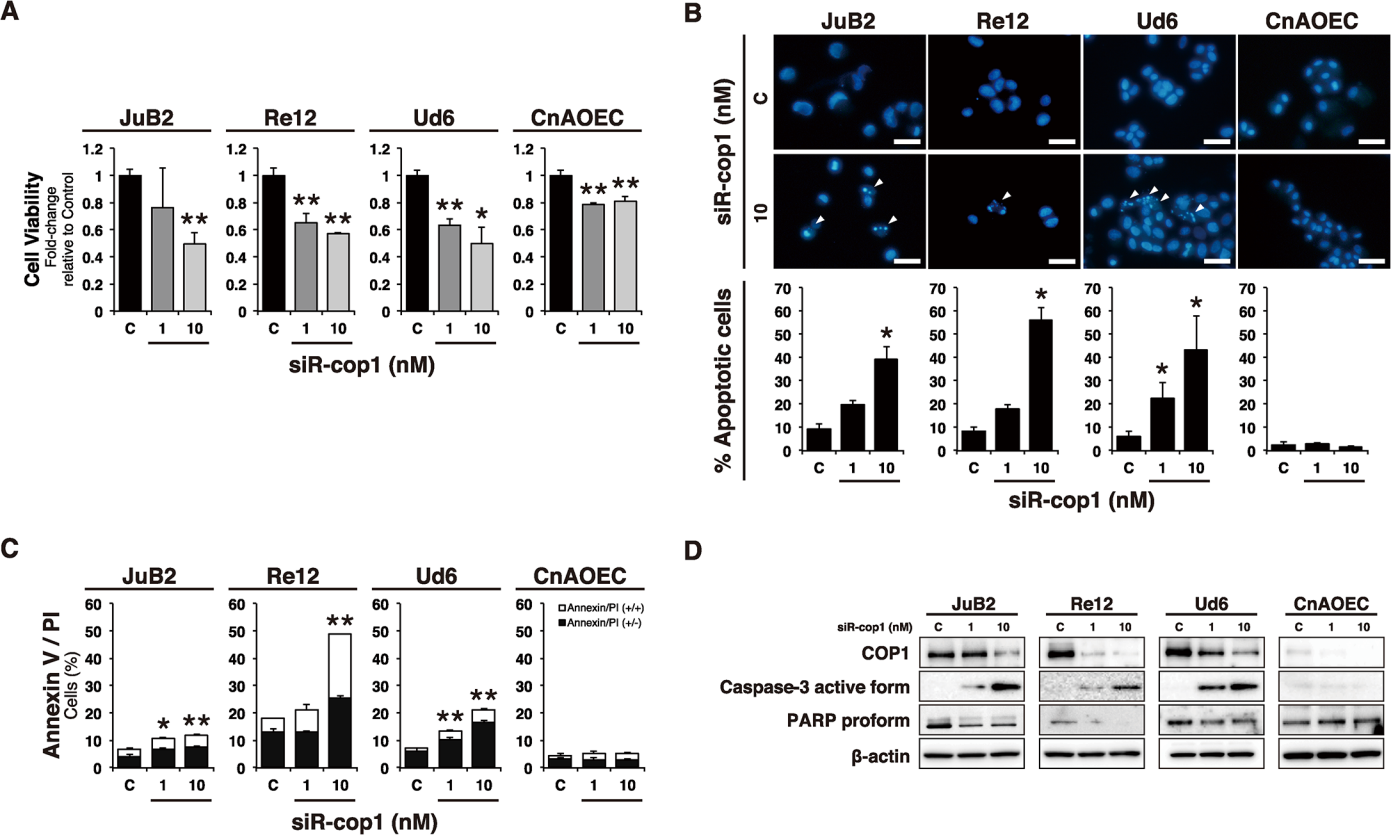
COP1 knockdown decreased the number of viable cells and induced apoptosis in HSA cell lines just as did miR-214-transfection. (A) Cell viability was assessed by performing the MTT assay. COP1 knockdown by siRNA dose-dependently decreased the number of viable cells of all HSA cell lines. COP1 knockdown decreased the viable cells of control EC; however, the degree was slight compared to that of HSA cell lines. All data are presented as the mean of triplicate experiments with error bars indicating the s.d. (Two-tailed t-test; *p<0.05, **p<0.01). (B) Examination of nuclear morphology by use of Hoechst 33342 stain. COP1 knockdown increased the number of cells with fragmented nuclei (white arrow heads) dose-dependently among cells of all HSA cell lines just as did miR-214-transfection. However, these apoptotic changes were not observed in siR-cop1 transfected control EC. Scale bars in the photographs indicate 25 μm. The graph shows the percentage of cells with fragmented nuclei among a total of 500 cells. All data are expressed as the mean of triplicate experiments with error bars indicating the s.d. (Two-tailed t-test; *p<0.05). (C) Apoptotic cell count by Annexin V/ PI double staining. Annexin V positive/ PI negative and Annexin V positive/ PI positive cells represent early and late-phase apoptosis, respectively. COP1 knockdown increased both early and late-phase apoptosis in HSA cell lines as same manner as miR-214-transfection but not in control EC. All data are present as the mean of triplicate experiments with error bars indicating the s.d. The statistical significances stated were referred to the entire Annexin/PI population (Two-tailed t-test; *p<0.05, **p<0.01). (D) Immunoblotting for caspase-3 active form and PARP proform. COP1 knockdown increased the amounts of active-form of caspase-3 and cleavage of PARP proform in HSA cell lines as same manner as miR-214-transfection but not in control EC. β-actin was used for the normalization of the amount of sample loaded.

**Fig 7:**
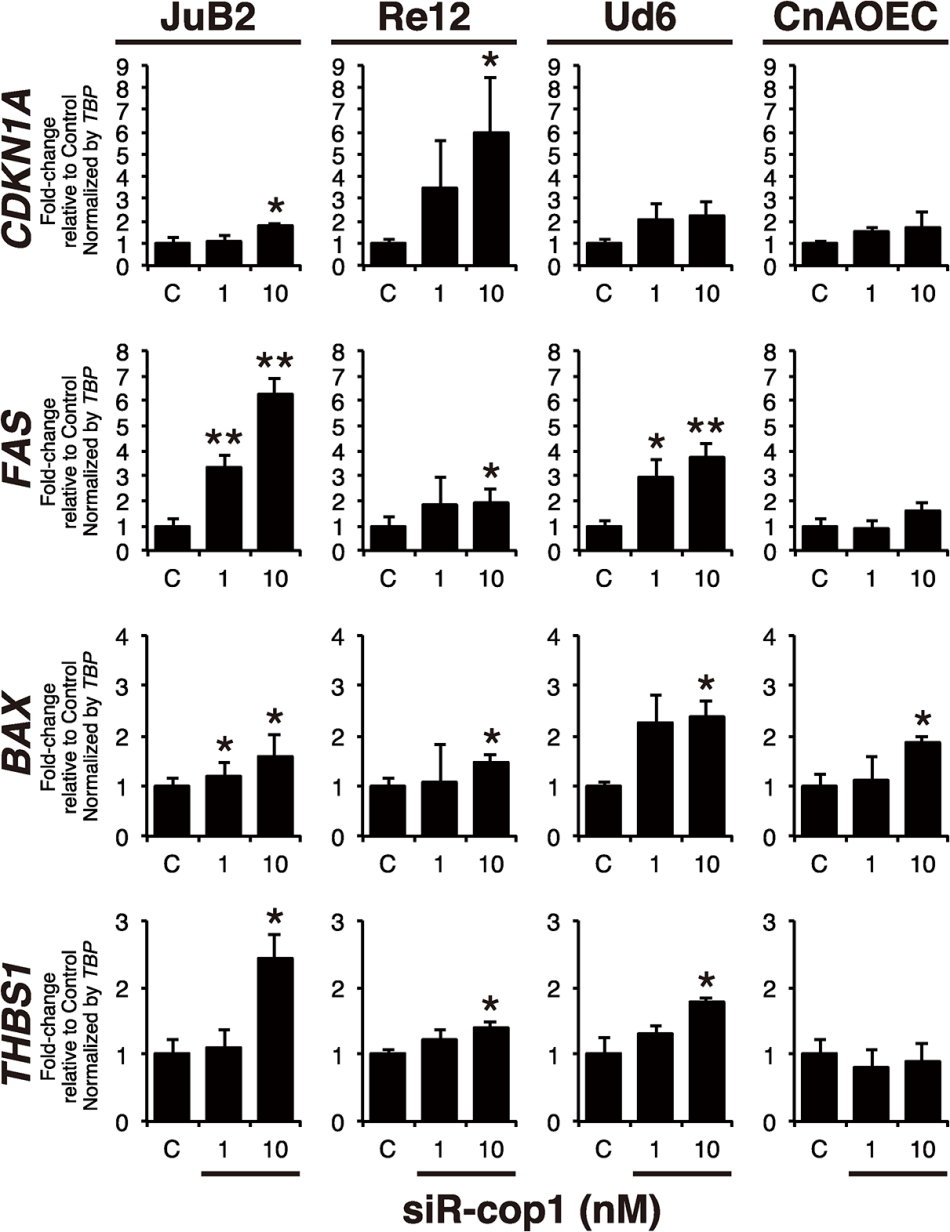
COP1 knockdown provoked expression of p53-regualted genes expressions just as did miR-214-transfection. Expression of p53-regulated genes including *CDKN1A*, *FAS*, *BAX* and *THBS1* in siR-cop1 transfected samples assessed by qRT-PCR. The expression of p53-regulated genes was up-regulated dose-dependently in all HSA cell lines transfected with 1 nM or 10 nM of siR-cop1 as same manner as miR-214-transfection although siR-cop1 slightly or hardly increased these p53-regulated genes. All data are presented as the mean of triplicate experiments with error bars indicating the s.d. (Two-tailed t-test; *p<0.05, **p<0.01).

Our results showed that miR-214 induced apoptosis and that miR-214 directly targeted COP1, which was overexpressed in HSA cell lines. Next, to confirm whether *COP1* knockdown could result in apoptosis in HSA cells, we conducted specific knockdown of COP1 by using its siRNA (siR-cop1). The results indicated that *COP1* knockdown decreased the number of viable cells (Fig 6A), but increased the number of apoptotic cells (Fig 6B, 6C and 6D) and up-regulated the expression of the p53-regulated genes in the HSA cell lines (Fig 7). However, siR-cop1 induced slight growth inhibition without apoptosis or significant activation of p53-regulated genes except *BAX* in control EC similar to miR-214-transfection in control EC (Figs 6A, 6B, 6C, 6D and 7). Thus, *COP1* knockdown induced apoptosis in HSA cells by up-regulating p53-regulated gene expression, similar to the results obtained using miR-214-transfection. These results suggest that COP1 was an essential molecule responsible for miR-214-mediated apoptosis in HSA cells.

### miR-214-COP1 axis elicited apoptosis through p53

**Fig 8:**
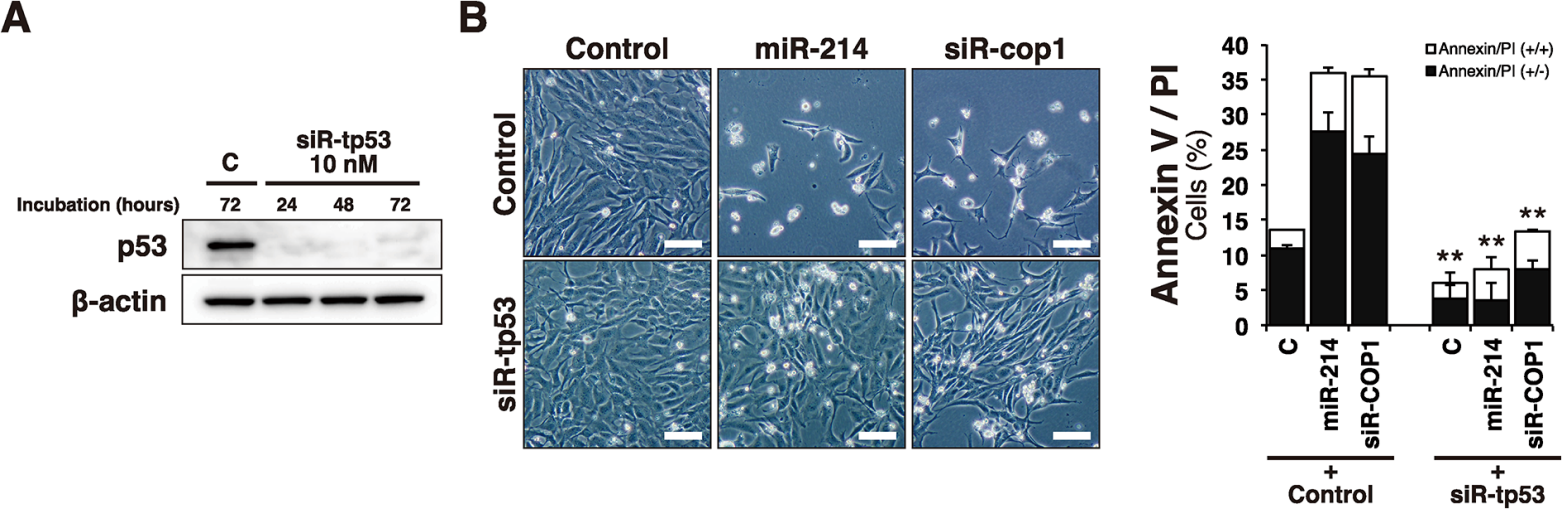
p53 knockdown by its siRNA abolished the apoptotic effects of miR-214-transfection and COP1 knockdown. (A) 10 nM of newly designed siR-tp53 successfully knockdowned the expression of p53 protein from 24 hours up to 72 hours post-transfection in Ud6 cell line. (B) p53 knockdown by siR-tp53 abolished the effect of apoptotic induction in Ud6 cells. Co-transfection of Ud6 cells with miR-214 or siR-cop1 and control RNA induced apoptosis; however, when siR-tp53 replaced the control RNA, apoptosis was hardly or only slightly induced in the cells. Scale bars in the photographs indicate 100 μm. All final concentrations of small RNA transfection were 10 nM in this examination. All data are present as the mean of triplicate experiments with error bars indicating the s.d. The statistical significances stated were referred to the entire Annexin/PI population (Two-tailed t-test; *p<0.05, **p<0.01).

**Fig 9:**
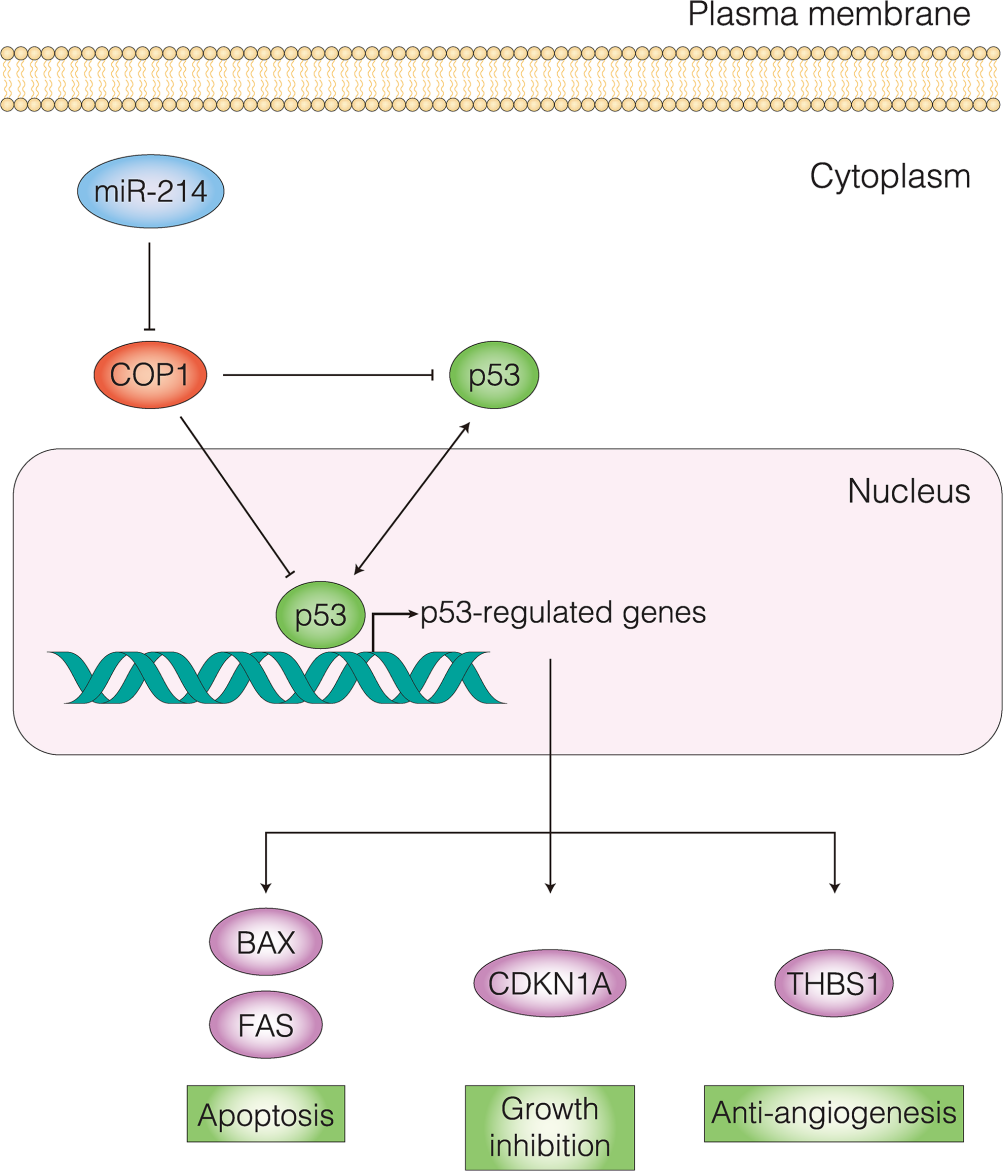
Schematic diagram of miR-214-COP1-p53 axis in HSA. COP1 contributes to proteasomal degradation of p53, thereby, inhibiting the expression of p53-regulated genes such as *BAX*, *FAS*, *CDKN1A* and *THBS*, which are activated by p53. This loss of expression of these genes results in inhibition of apoptosis, growth inhibition and anti-angiogenesic action. As miR-214 directly targets COP1, the up-regulation of miR-214 provokes apoptosis by inhibiting COP1-dependent degradation of p53.

As *COP1* knockdown up-regulated the expression of p53-regulated genes, we hypothesized that COP1 controlled p53-regulated genes through its interaction with p53. To investigate the correlation with p53 and miR-214-COP1-mediated apoptosis, we examined whether *p53* knockdown could affect the miR-214-COP1-mediated apoptosis. First, we newly designed an siRNA specific for p53 (siR-tp53) and assessed whether siR-tp53 could appropriately repress the expression of p53 protein. As a consequence, we confirmed that siR-tp53 successfully down-regulated the expression of p53 protein from 24 hours up to 72 hours after Ud6 cells had been transfected with it (Fig 8A). Next, in order to clarify the relationship between p53 and the miR-214-COP1 axis, we introduced miR-214 or siR-cop1 alone or with knockdown of *p53* by siR-tp53 in Ud6 cells. As a result, the single treatment with miR-214 or siR-cop1 induced apoptosis in these cells; however, miR-214 or siR-cop1 treatment of cells subjected to *p53* knockdown failed to induce apoptosis in Ud6 cells (Fig 8B). The result that *p53* knockdown abolished miR-214-COP1-mediated apoptosis indicates that p53 played an indispensable role in the apoptosis caused by the introduction of miR-214 or the knockdown of COP1. Thus, we demonstrated that p53 was the molecule responsible for miR-214-COP1-mediated apoptosis and up-regulation of p53-regulated genes (Fig 9).

Thus, our experiments indicated that miR-214 expression was down-regulated in HSA cells as well as in clinical samples from dogs with HSA and that the restoration of its expression by transfection of HSA cells with synthetic miR-214 resulted in apoptosis in these cells. This apoptosis occurred through p53-regulated gene activation as a consequence of the direct targeting of COP1, a negative regulator of p53, by miR-214.

## Discussion

miR-214 functions as an anti-oncomir in various tumors [17,22,26,30] and also negatively regulates angiogenesis [16,32]. However, the function of miR-214 in malignant endothelial proliferative diseases had not yet been reported. In this study, we examined miR-214 expression in HSA cell lines and its function by introducing miR-214 into HSA cells. Here, we identified miR-214 as a novel anti-oncomir, which was down-regulated in both clinical samples and cell lines of HSA. We identified COP1 as a novel conserved target of miR-214, which was an oncogene overexpressed in HSA cell lines. Furthermore, we demonstrated that miR-214 induced apoptosis by directly targeting *COP1*, thereby positively regulating p53 transcriptional activity in HSA cell lines.

The survival and functional regulations of endothelial cells are controlled by various molecules including miRNAs [14]. miR-214 is highly expressed in main vascular types of endothelium and controls angiogenesis negatively by blocking the release of angiogenic growth factors such as VEGF, bFGF and PDGF [16]. Presently, we found that miR-214 was significantly down-regulated in clinical samples of splenic HSA and in HSA cell lines, suggesting that nullification of miR-214 in HSA could contribute to continuously activated angiogenesis, and subsequent accelerated growth of neoplastic endothelial cells. Furthermore, we showed that THBS1 (thrombospondin), which is a strong angiogenesis inhibitor, was induced by miR-214 in HSA cell lines. Previously, Mil *et al*. reported that miR-214 is able to increase THBS1 expression in EC [16]; however, the mechanism still remains unclear. Judging from our results that miR-214 controlled p53-regulated genes including THSB1, miR-214 might control angiogenesis by targeting not only growth factor release but also THBS1 in a p53-dependent manner in HSA. Based on these reports, our findings suggest that down-regulation of miR-214 is one of the key genetic events leading to tumorigenesis and continuous angiogenesis in HSA.

Inhibition of apoptosis is critical for tumorigenesis and tumor progression. Recent studies revealed that the down-regulation of certain miRNAs is responsible for inhibition of apoptosis in various cancers [50]. miR-214 is one of the miRNAs that induces apoptosis and is down-regulated in various tumors including cervical cancer [19,51], hepatocellular carcinoma [20], glioma [25], rhabdomyosarcoma [30], bladder cancer [28], and colon cancer [24]. For functional assessment of miR-214, we transfected HSA cell lines with this miRNA. Consistent with our hypothesis, transfection of these cells with miR-214 induced typical apoptotic changes such as nuclear fragmentation, an increase in the number of Annexin-V positive cells, activation of caspase-3 and cleavage of the PARP proform in HSA cell lines. Although we failed to determine whether intrinsic or extrinsic apoptosis signaling was induced by miR-214 because of the lack of appropriate antibodies specific for canine molecules, we found that miR-214 increased the expression of direct transcriptional targets of p53 such as *CDKN1A*, *FAS*, *BAX* and *THBS1*; and, therefore, p53 was suggested to play important roles in miR-214-induced apoptosis.

Inactivation of p53 is the key event in most of malignancies, and mutation of p53 has been thought to be responsible for tumorigenesis in most cancers [34]; however, recent studies showed that functional inactivation of p53 by alteration of proteasomal p53 regulators such as MDM2 also contributes to escape from p53-regulated growth arrest and cell death. In fact, p53 mutations are rare in both HSA and AS [36,37], and p53 is still retains its wild-type form in the majority of HSA and AS cases, even though transcriptional activation by it is inhibited [39]. Therefore, we explored the possibility that miR-214 targeted some molecule regulating p53. From the result of computer target prediction of miR-214, we hypothesized that COP1 E3 ubiquitin protein ligase, which is a critical negative regulator of p53 as well as MDM2, would be a possible conserved target of miR-214. We considered that the action of this E3 ubiquitin ligase would possibly correlate with the functional dysregulation of p53 in HSA. As we expected, miR-214 was able to bind to a COP1 3'UTR site; and treatment of HSA cells with miR-214 down-regulated the expression of both COP1 mRNA and its protein. Furthermore, we found the overexpression of COP1 and the specific knockdown of COP1 by its siRNA induced apoptosis and recovered p53 transcriptional activity in HSA cells just like miR-214. Moreover, co-knockdown of p53 by its siRNA abolished miR-214-COP1-mediated apoptosis, indicating that p53 is the molecule responsible for the miR-214-COP1 mediated apoptosis. Our results showing that miR-214 was capable of inducing apoptosis to HSA cells by targeting COP1 suggest that miR-214 down-regulation, as well as COP1 overexpression, play essential roles in functional p53 inactivation and apoptotic inhibition in HSA cells. The miR-214-COP1-p53 axis was likely to be inactivated and relate to tumorigenesis in dogs with HSA. The importance of miR-214 and COP1 expression in HSA suggests that this miR-214-COP1-p53 axis could likely be an effective target and recovering the function of inactivated p53 is a key to establish novel therapeutics for HSA.

In conclusion, we demonstrated that miR-214 played essential roles in the regulation of apoptosis by targeting *COP1* in HSA. miR-214 down-regulation and COP1 overexpression might be one of the cause of p53 dysregulation in HSA. Hence, the discovery that miR-214 regulated p53 signals by targeting COP1 provides a new important target to block the inhibition of apoptosis, and it may well contribute to the development of novel therapeutics for malignant endothelial proliferative diseases.

## Supporting Information

S1 Fig Computer prediction by TargetScan and miRDB. (A) Computer target and target site prediction of miR-214 in dogs by TargetScan 6.2. COP1 mRNA target sites of miR-214 are conserved among species including human and canine and show high affinity for miR-214. (B) Computed target and target site prediction of miR-214 in dogs by miRDB. Target sites are conserved to the same extent as shown with TargetScan 6.2.
(TIF)

S2 Fig miR-214 expression levels of HSA cell lines and CnAOEC after miR-214-transfection. miR-214-transfection successfully increased intracellular expression of miR-214 in all cell lines used in this study although the efficiency differed in each cell line. The intracellular miR-214 levels after transfection did not correspond to the degree of decreased viable cells and induction of apoptosis, indicating that the responses to miR-214-transfection was not dependent on the efficiency of transfection but the primary levels of miR-214 or COP1 expression.
(TIF)

S1 Table Splenic HSA and normal splenic tissues for miRNA expression analysis. Information on dogs as the source of tissue samples used in this research.
(TIF)

S2 Table PCR primers used in this study. Detailed information on PCR primers. All primers were newly designed by the authors.
(TIF)
