# Supplementary figures and images for: MicroRNA-214 Promotes Apoptosis in Canine Hemangiosarcoma by Targeting the COP1-p53 Axis
Source: PLoS One. 2015 Sep 3;10(9):e0137361. doi: 10.1371/journal.pone.0137361 (PMC4559432; doi:10.1371/journal.pone.0137361)

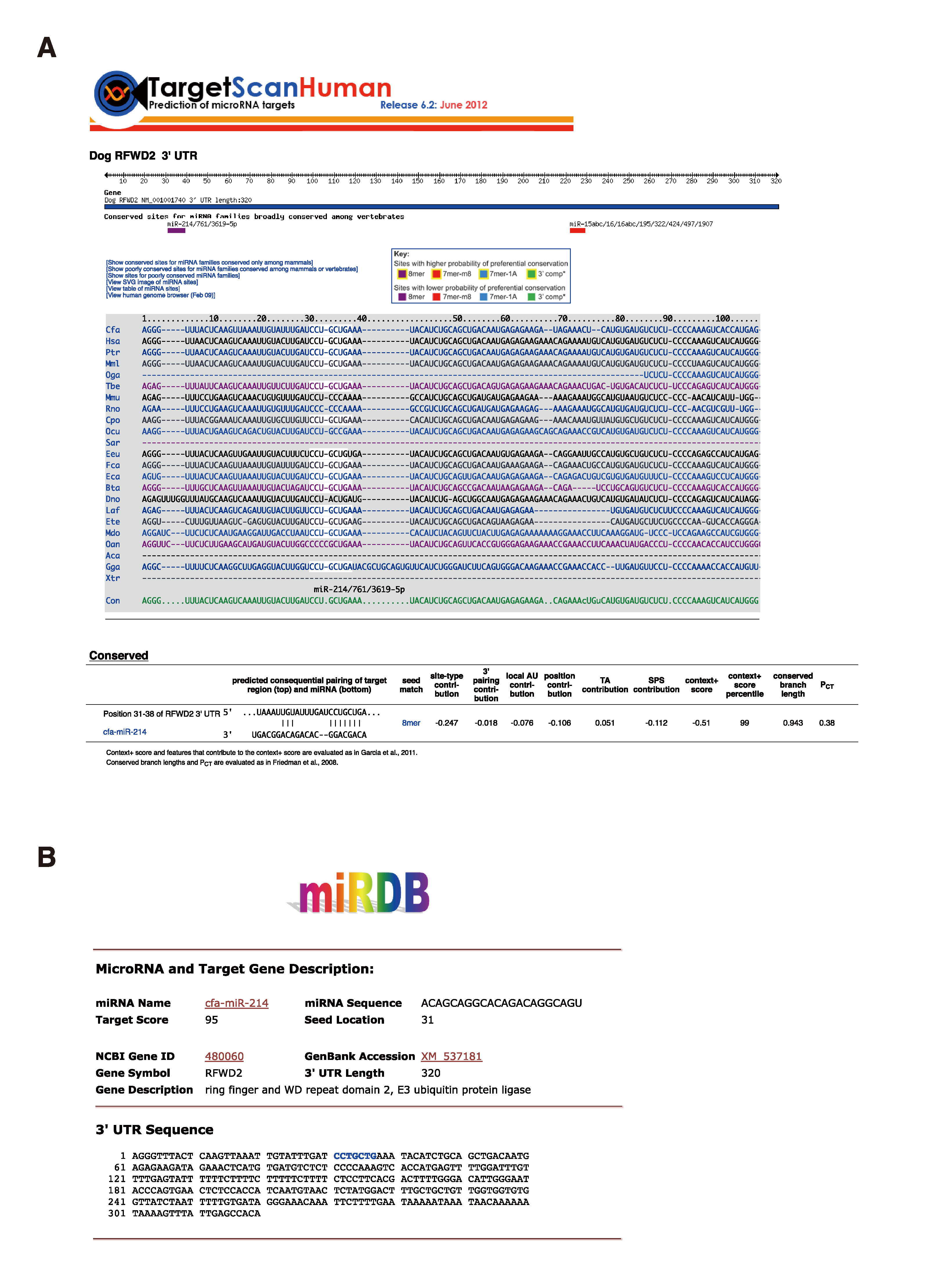

Supplement: S1 Fig — (A) Computer target and target site prediction of miR-214 in dogs by TargetScan 6.2. COP1 mRNA target sites of miR-214 are conserved among species including human and canine and show high affinity for miR-214. (B) Computed target and target site prediction of miR-214 in dogs by miRDB. Target sites are conserved to the same extent as shown with TargetScan 6.2. (TIF) [file pone.0137361.s001.tif]

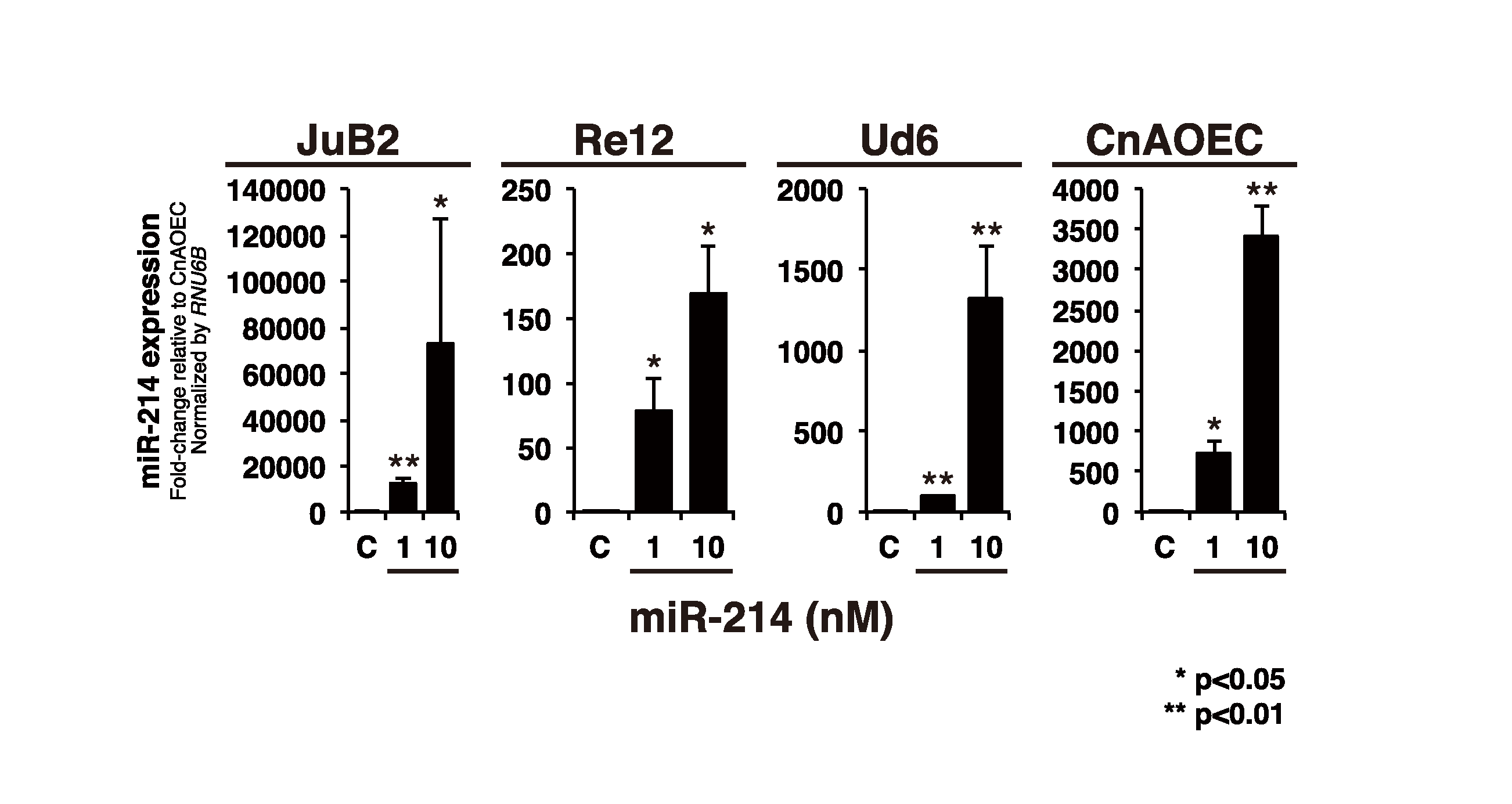

Supplement: S2 Fig — miR-214-transfection successfully increased intracellular expression of miR-214 in all cell lines used in this study although the efficiency differed in each cell line. The intracellular miR-214 levels after transfection did not correspond to the degree of decreased viable cells and induction of apoptosis, indicating that the responses to miR-214-transfection was not dependent on the efficiency of transfection but the primary levels of miR-214 or COP1 expression. (TIF) [file pone.0137361.s002.tif]

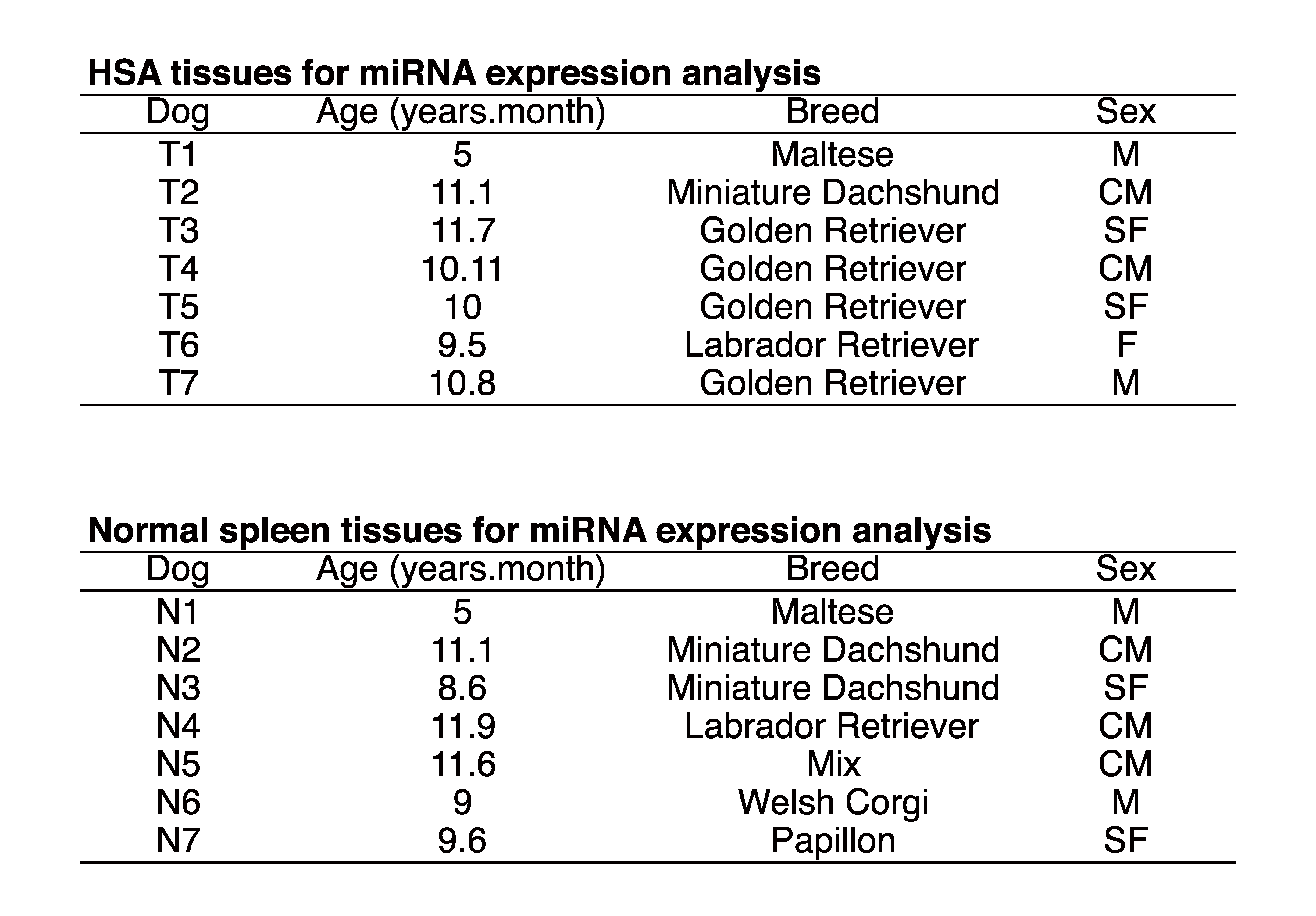

Supplement: S1 Table — Information on dogs as the source of tissue samples used in this research. (TIF) [file pone.0137361.s003.tif]

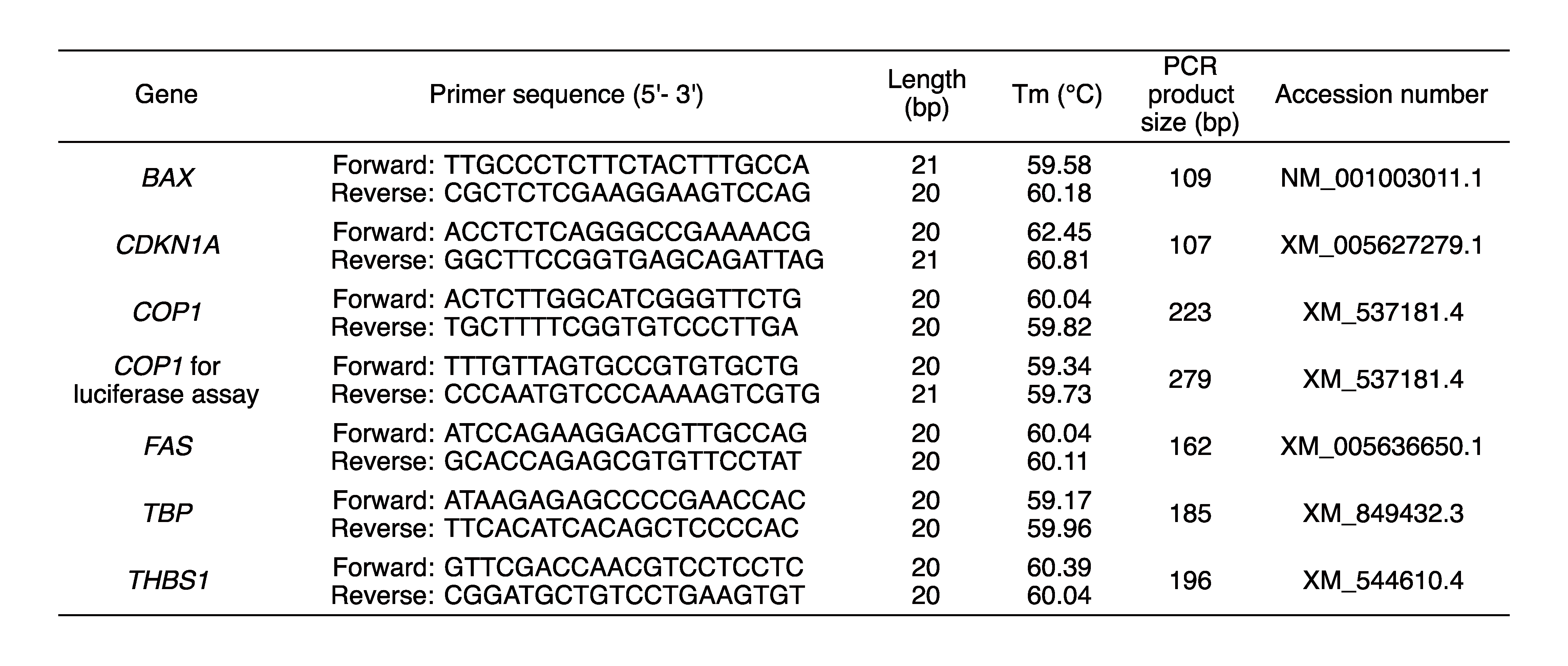

Supplement: S2 Table — Detailed information on PCR primers. All primers were newly designed by the authors. (TIF) [file pone.0137361.s004.tif]
